# Metabolic control of neuroinflammation: focus on itaconate and its derivatives in CNS disorders

**DOI:** 10.3389/fimmu.2026.1726379

**Published:** 2026-02-06

**Authors:** Ying Wang, Shihui Liu, Weijie Zhu, Pengyu Hao, Jiacan Xu, Diqi Mai, Ran Chen, Haojie Han, Xuechen Bian, Bodong Wang

**Affiliations:** 1 Department of Neurosurgery, the 960th Hospital of PLA (General Hospital of Jinan Military Command), Jinan, Shandong, China; 2 School of Clinical Medicine, Shandong First Medical University, Jinan, Shandong, China; 3 School of Clinical Medicine, Shandong Second Medical University, Weifang, Shandong, China; 4 Department of Histology and Embryology, School of Basic Medical Sciences, Cheeloo College of Medicine, Shandong University, Jinan, China

**Keywords:** central nervous system diseases, itaconate, metabolic control, microglia, neuroinflammation

## Abstract

The activation of microglia, which are the resident immune cells of the central nervous system (CNS), underpins the pathogenesis of neuroinflammatory and neurodegenerative diseases. Metabolic reprogramming has recently been recognized as a critical mechanism that regulates microglial activation because distinct activation phenotypes are tightly coupled to specific metabolic profiles that shape their functional and inflammatory responses. Accumulating evidence indicates that microglia produce itaconate through the tricarboxylic acid cycle, and itaconate and its derivatives play key antioxidant and anti-inflammatory roles. Mechanistically, itaconate has a major impact on the metabolic processes and functional state of microglia by blocking the NF-κB signaling route, activating the Nrf2 signaling pathway, and inhibiting succinate dehydrogenase synthesis as well as NLRP3 inflammatory vesicle activation. Collectively, these actions confer significant protection against CNS disorders, including ischemic stroke, Alzheimer’s disease, Parkinson’s disease, and cerebral hemorrhage. Furthermore, structurally optimized itaconate derivatives exhibit enhanced pharmacokinetics and bioactivity. This review highlights the pivotal role of itaconate and its derivatives in microglial regulation, explores their therapeutic potential in neurological diseases, and outlines future research directions, with the aim of providing a theoretical foundation for novel metabolic interventions.

## Graphical Abstract

**Figure:**
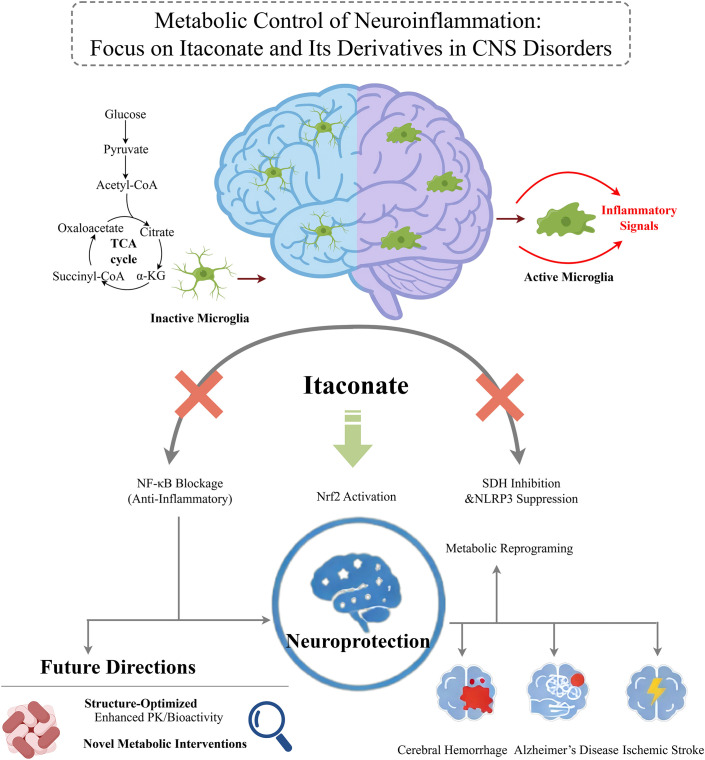
Metabolic control of neuroinflammation by itaconate. Itaconate reprograms microglial metabolism to exert neuroprotection. This multifaceted action involves inhibiting NF-κB and NLRP3, activating Nrf2, and suppressing succinate dehydrogenase (SDH). Structurally optimized itaconate derivatives thus emerge as promising metabolic interventions for cerebral hemorrhage, Alzheimer’s disease, and ischemic stroke, highlighting the therapeutic potential of targeting the immunometabolic crosstalk.

## 1 Introduction

As resident macrophages of the central nervous system (CNS), microglia are the primary immune cells of the CNS (1). They regulate inflammatory responses by releasing key neuroinflammatory mediators such as cytokines, chemokines, and reactive oxygen species (ROS), and phagocytose pathogens and debris during injury, infection, or pathological changes (2). However, sustained or excessive activation of microglia can lead to chronic inflammation. This contributes to the development of neurodegenerative diseases, such as Alzheimer’s and Parkinson’s disease, underscoring the need to modulate microglial activation to prevent detrimental neuroinflammation (3).

Several studies have demonstrated that the metabolic properties of microglia regulate their activation and activity (4, 5). Under physiological conditions, resting microglia rely primarily on the tricarboxylic acid (TCA) cycle and mitochondrial oxidative phosphorylation (OXPHOS) to efficiently generate ATP for their surveillance and housekeeping functions (6). However, upon exposure to pro-inflammatory stimuli, microglia undergo a metabolic switch; they accelerate glycolysis to generate energy and metabolic intermediates for pro-inflammatory responses while partially inhibiting the TCA cycle. This metabolic shift provides energy and generates key inflammatory mediators (7). TCA cycle metabolites, such as succinate, fumarate, and itaconate, play crucial roles in regulating the immune phenotype and inflammatory responses of macrophages (8). Among these, itaconate has recently become a research focus owing to its significant immunomodulatory properties.

Itaconate, an endogenous metabolite derived from the TCA cycle, has attracted increasing attention owing to its biological and industrial relevance. Physiologically, it is synthesized in mammalian cells through cis-aconitate decarboxylation, linking metabolic reprogramming to immune regulation (9–11). Beyond its endogenous generation, itaconate has long been recognized in the field of chemical engineering as a versatile platform compound, and is widely applied in the production of polymers, resins, and bio-based plastics owing to its unsaturated dicarboxylic acid structure (12, 13). This dual identity highlights the significance of itaconate. However, its specific functions in the CNS remain unclear. It is broadly distributed among CNS cell types, including neurons and microglia, and is particularly relevant to microglial metabolism and activation (1, 14). Itaconate has emerged as a critical regulator of microglial inflammatory responses (15–19), highlighting its therapeutic potential in controlling neuroinflammation. However, its direct application is hindered by poor blood–brain barrier (BBB) penetration, which limits effective CNS delivery (20). To address this limitation, a series of itaconate derivatives with enhanced stability and permeability have been developed, many of which have demonstrated superior anti-inflammatory effects in preclinical models (10, 20, 21). Collectively, these findings emphasize the translational promise of itaconate-based compounds in CNS disorders, highlighting the necessity of optimized derivatives for future therapeutic use.

This review focuses on the latest molecular mechanisms through which itaconate and its derivatives regulate metabolic reprogramming and microglial activation. Based on this, we systematically elucidated their roles in various CNS diseases and proposed emerging research directions in this field. By integrating cutting-edge evidence, this study aimed to comprehensively elucidate the functional framework of itaconate in the CNS, providing a theoretical basis for the development of therapeutic strategies targeting the itaconate pathway in neurological diseases.

## 2 Synthesis and biological function of itaconate

Itaconate is an α,β-unsaturated dicarboxylic acid with two electrophilic groups and a conjugated double bond (22). This structure makes it a potent Michael acceptor and a crucial precursor in chemical synthesis because of its ability to modify cysteine residues and undergo addition, esterification, and polymerization reactions. Itaconate plays a role in the immunological response of macrophages, as evidenced by the discovery in 2011 that it is generated via the decarboxylation of cis-aconitate in the TCA cycle and accumulates significantly in LPS-activated macrophages (23). Michelucci et al. discovered that LPS-activated macrophages exhibit substantial upregulation of the immune response gene 1 (*Irg1*). The itaconate synthesis mechanism in macrophages involves cis-aconitate decarboxylase (CAD, also known as aconitate decarboxylase 1, ACOD1), which is encoded by *Irg1* and catalyzes the synthesis of itaconate from cis-aconitate (24). Itaconate is catabolized in the mitochondria, mirroring its site of synthesis. Succinyl-CoA synthetase converts itaconate to itaconyl-CoA, which is then transformed into citramalyl-CoA by methylglutaconyl-CoA hydratase. Citramalyl-CoA is subsequently cleaved into pyruvate and acetyl-CoA by the citrate lyase subunit beta-like (CLYBL) into pyruvate and acetyl-CoA (25). These metabolites reenter the TCA cycle for energy metabolism. This pathway not only sustains cellular energy metabolism, but also provides essential regulatory mechanisms for the immune response (24, 26).

Building on its defined metabolic synthesis and catabolic pathways, subsequent studies have revealed that itaconate functions not only as a metabolic byproduct, but also as a critical regulator of immune and inflammatory processes. In 2016, researchers first demonstrated that itaconate inhibits succinate dehydrogenase (SDH) in macrophages, thereby suppressing inflammation (18). Subsequent studies have revealed its ability to mitigate inflammatory tissue damage through two mechanisms: regulation of mitochondrial activity and reduction of ROS generation (10, 27). Furthermore, studies have demonstrated that itaconate and its derivatives can improve cellular self-defense mechanisms by activating the Nuclear factor erythroid 2-related factor 2 (Nrf2) pathway (10, 28, 29) while concurrently suppressing NF-κB-mediated microglial activation and pro-inflammatory gene expression (29). This coordinated modulation of redox and inflammatory signaling underpins their crucial antioxidant and anti-inflammatory functions.

Furthermore, recent studies have refined the functional distinction among ACOD1, endogenous itaconate, and itaconate derivatives (30, 31). ACOD1 is increasingly being recognized as an active immunometabolic regulator, rather than merely an enzyme for itaconate biosynthesis, and its induction reflects coordinated metabolic reprogramming in activated myeloid cells and influences inflammatory and redox-related signaling through both itaconate-dependent and -independent mechanisms (31). In contrast, the biological effects of endogenous itaconate appear to be highly context-dependent. Although intracellular itaconate accumulation commonly accompanies ACOD1 induction, its contribution to downstream signaling events, including Nrf2-associated responses, varies between cellular states and experimental conditions. Notably, many robust anti-inflammatory and cytoprotective effects attributed to the itaconate pathway have been consistently demonstrated using cell-permeable and electrophilic itaconate derivatives, such as dimethyl itaconate (DMI) and 4-oxoicarboxylic acid (4-OI), which enable the efficient engagement of downstream signaling pathways (30). These observations provide an important conceptual basis for the introduction and discussion of itaconate derivatives in greater detail in the following section.

## 3 Similarities and differences between itaconate and its derivatives

Building on the context-dependent effects of endogenous itaconate, numerous studies have demonstrated that the structural modification of itaconate generates a series of derivatives with enhanced cell membrane permeability, increased lipid solubility, and improved tissue stability (32). These physicochemical properties facilitate reliable cellular uptake and sustained intracellular activity, thereby enabling robust interrogation of itaconate-associated immunometabolic pathways (33, 34). Accordingly, itaconate derivatives have been widely employed in experimental systems, and emerging evidence indicates that certain derivatives can influence metabolic reprogramming in microglia and modulate inflammatory responses, highlighting their functional relevance in neuroimmunological research (**Table 1**).

**Table 1:** Comparison of itaconate and its derivatives.

Category	Attribute	Itaconate	Derivatives (e.g., 4-OI, DMI)	References
Physicochemical Properties	Solubility & Stability	Highly hydrophilic; less chemically stable.	Lipophilic (high lipid solubility); chemically stable (especially DMI).	(35–37)
	Permeability	Low membrane permeability (transport-dependent entry).	High cell membrane permeability.	(10, 20, 35)
Mechanism of Action	Nrf2 Pathway Activation	Activates Nrf2 via KEAP1 alkylation (commonality).	Activates Nrf2 via KEAP1 alkylation (commonality).	(10, 16, 19, 38)
	SDH Inhibition	Potent inhibitor (competitive inhibition of succinate dehydrogenase)	Weak/indirect inhibitor (4-OI lacks direct SDH inhibition; DMI may hydrolyze)	(18, 19, 39)
	Inflammation Modulation	Inhibits NF-κB, NLRP3, and glycolysis.	Potently inhibits NF-κB, NLRP3, and glycolysis.	(16, 19, 29, 40)
Clinical Potential	Application Scope	Limited to *in vitro*/laboratory models due to poor BBB penetration.	High potential for *in vivo*/clinical use due to improved pharmacokinetics	(20, 36, 41, 42)

**Figure 1:**
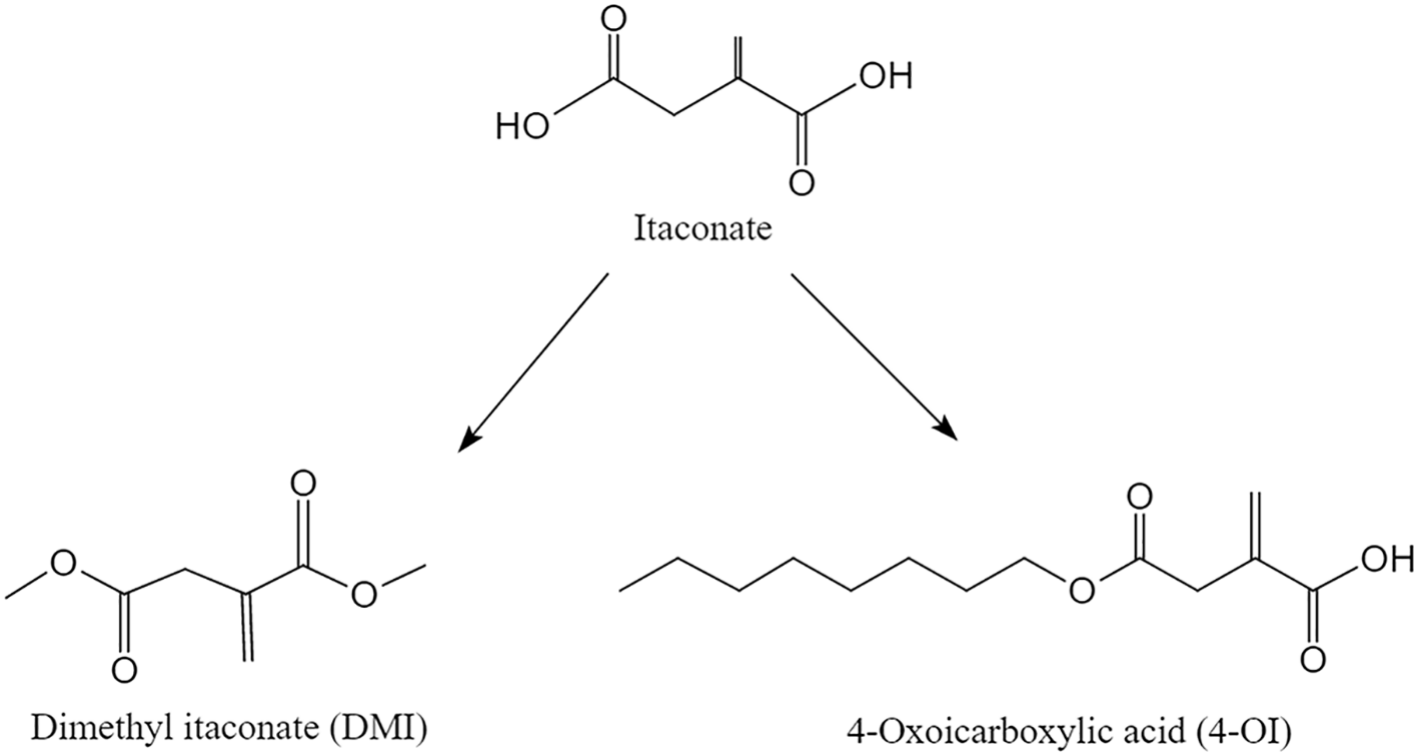
Chemical structures of itaconate and its derivatives, 4-octyl itaconate (4-OI) and dimethyl itaconate (DMI). Itaconate: An α, β-unsaturated dicarboxylic acid with a conjugated double bond and two electrophilic groups. 4-OI: Derivative featuring a carbonyl group introduced at the 4-position of the itaconate skeleton. DMI: Diester formed by the dimethyl esterification of itaconate, replacing carboxyl hydrogens with methyl groups.

4-OI is generated by introducing a carbonyl group at the 4-position of the itaconate skeleton (**Figure 1**). 4-OI exhibits potent tissue-targeting, high cell membrane permeability, enhanced lipid solubility, and broad tissue distribution (35). Via the enolization-mediated modification of IκBζ and kelch-like each-related protein 1 (KEAP1), it can inhibit the pro-inflammatory NF-κB pathway and activate the anti-inflammatory Nrf2 pathway (16, 19). Furthermore, 4-OI contributes to metabolic homeostasis by suppressing excessive glycolysis, reducing oxidative stress, and maintaining the cellular antioxidant status. These effects are achieved, at least partially, by boosting NADPH and glutathione (GSH) synthesis (19, 43).

DMI is synthesized via the dimethyl esterification of the carboxyl groups of itaconate. This modification replaces the carboxyl hydrogens with methyl groups. DMI enhances metabolic stability and modulates pro-inflammatory metabolic reprogramming in microglia. Its mechanism involves the suppression of the ROS, HIF-1α, and glycolytic pathways, while also activating the Nrf2 pathway through KEAP1 enolization (38). Reported in 2019, DMI significantly inhibits the pathological transition of peri-infarct microglia, suggesting its potential as a therapeutic agent against ischemic cerebral infarction (36). Furthermore, a study from 2020 demonstrated that DMI reduced disease severity in a chronic C57BL/6 experimental autoimmune encephalomyelitis (EAE) model by attenuating microglial activation (37) (**Figure 1**).

## 4 Effects of itaconate and its derivatives on microglia

Microglia are immune cells of the CNS that play important surveillance and protective roles. They regulate neuronal activity by sensing environmental changes (e.g., injury, pathogen infection, or neurodegenerative lesions) to initiate an immune response (44). Under physiological conditions, microglia maintain a surveillance (“resting”) state. However, exposure to pathological stimuli (e.g., injury or infection) triggers rapid activation, characterized by morphological transformation into an amoeboid phenotype with enhanced phagocytic capacity and increased production of immune mediators (45). The activated microglia release cytokines, chemokines, and other inflammatory factors that orchestrate neuroinflammation (46). Interactions between activated microglia and damaged neurons influence both pathological progression and repair mechanisms within an injured CNS (18). Notably, inflammatory stimuli induce *Irg1* expression, which catalyzes itaconate production via the TCA cycle (47).

Accumulating evidence has demonstrated that itaconate inhibits microglial activation and attenuates neuroinflammation. Furthermore, experimental evidence that has accumulated in recent years has consistently demonstrated that DMI exerts a potent inhibitory effect on microglial activation, both *in vitro* and *in vivo*. In cultured primary microglia, DMI treatment significantly reduced the proportion of CD80^+^ cells, a key surface marker of microglial activation, as determined using flow cytometry. In parallel, *in vivo* DMI administration to EAE mice markedly decreases the density of Iba1^+^ microglia in the spinal cord, accompanied by the attenuation of neuroinflammation and disease severity (37). Consistent reductions in microglial activation have been further observed in the spinal cord, where DMI administration significantly decreases Iba1 fluorescence intensity and reduces the number of hypertrophic activated microglia in the dorsal horn (48). Beyond autoimmune models, DMI also mitigates high-fat diet (HFD)-induced neuroinflammation, restoring hippocampal microglia toward a homeostatic phenotype characterized by a reduced abundance of Iba1-positive cells (49). The anti-inflammatory activity of itaconate has also been demonstrated in neurodegenerative disease models. In a Parkinson’s disease (PD) model, itaconate treatment was found to significantly lower Iba1 protein expression and reduce the number of Iba1-positive microglia within the substantia nigra, as demonstrated by western blotting and immunohistochemical analyses (50). Similarly, in APPswe/PS1ΔE9 (APP/PS1) mice, DMI reduces microglial coverage surrounding amyloid plaques and promotes a phenotypic shift from the pro-inflammatory M1 state (CD86^+^, iNOS^+^) toward the anti-inflammatory M2 state (CD206^+^, Arg1^+^), an effect mediated partially through the activation of the Nrf2 signaling pathway (41).

In addition to suppressing microglial activation, itaconate and its derivatives play critical roles in regulating microglial polarization. Microglia dynamically transition between the pro-inflammatory M1 phenotype—typically induced by LPS or IFN-γ and characterized by robust cytokine production—and the anti-inflammatory M2 phenotype, which is promoted by IL-4 or IL-13 and supports tissue repair and inflammation resolution (51). In line with this paradigm, DMI administration significantly reduces peri-infarct M1-type microglia and lowers IL-1β protein levels in transient middle cerebral artery occlusion (tMCAO) mice, resulting in improved neurological outcomes (36). Moreover, DMI facilitates the M1-to-M2 phenotypic transition in LPS- and ATP-stimulated microglia (52), a finding further corroborated by the evidence that itaconate suppresses M1 polarization while promoting M2 polarization in microglial cells (53).

Itaconate and its derivatives, 4-OI and DMI, promote the transformation of microglia from the M1 to the M2 phenotype, not only through transient metabolic signaling, but also by establishing a robust ‘metabolic–epigenetic axis’ that stabilizes cell identity. This process intrinsically links metabolic reprogramming to chromatin remodeling (54). Mechanistically, the inhibition of SDH and the resulting metabolic shift regulate key epigenetic enzymes that suppress succinate-dependent histone demethylases (e.g., JMJD3) to epigenetically silence pro-inflammatory loci (e.g., IL-1b and TNF) (1, 55), whereas Nrf2 activation recruits histone acetyltransferases (HATs) to anti-inflammatory promoters (56). Crucially, redirected metabolism provides essential substrates for these modifications; accelerated glycolysis and fatty acid oxidation increase the intracellular pools of acetyl-CoA, lactate, and α-ketoglutarate (15). These metabolites fuel histone acetylation, novel histone lactylation (e.g., H3K18la), and TET-mediated DNA demethylation, respectively, which collectively maintain the open chromatin state of M2-associated genes (Arg1 and IL-10) (57, 58). This coordinated network, reinforced by the *Irg1*-itaconate feedback loop and STAT6 signaling, effectively locks microglia into a long-term neuroprotective M2 state (59, 60).

## 5 Specific molecular mechanisms of metabolic control of microglia activation by itaconate and its derivatives

**Figure 2:**
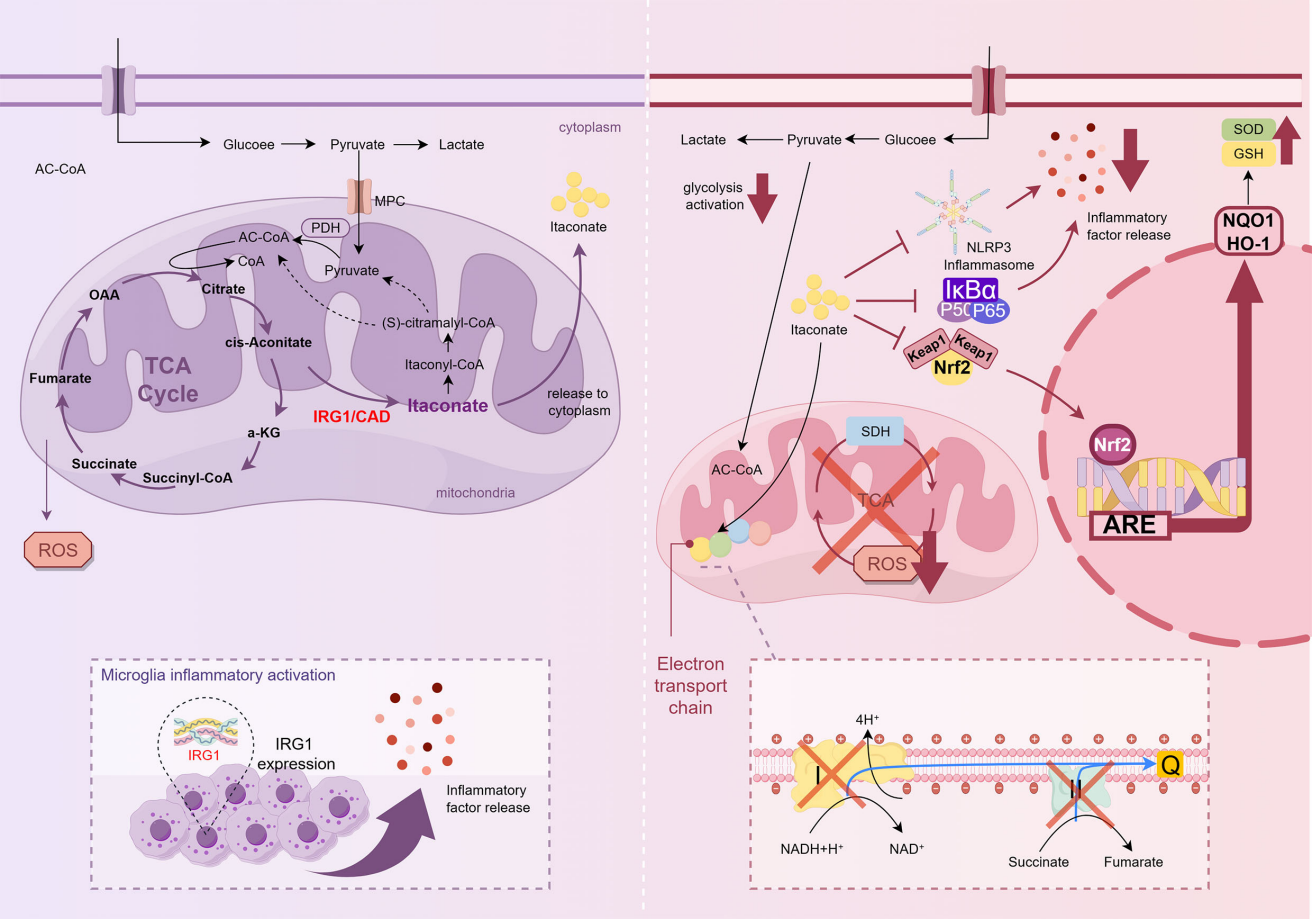
Itaconate synthesis and catabolic pathways. The *Irg1* gene, which encodes CAD, is markedly upregulated in inflammation-activated microglia. CAD catalyzes the decarboxylation of cis-aconitate into itaconic acid. The mitochondria subsequently catabolize itaconic acid through a multistep process, ultimately catabolizing itaconic acid to pyruvate and acetyl-CoA, which then re-enter the TCA cycle. Itaconic acid and its derivatives (e.g., itaconate) modulate microglial metabolic reprogramming through several key mechanisms. These include (1): suppression of SDH activity and consequent reduction in ROS production (2); inhibition of NLRP3 inflammasome activation (3); inhibition of the NF-κB signaling pathway; and (4) activation of the Nrf2 signaling pathway.

Rather than acting on a single target, itaconate and its derivatives exert a broad regulatory influence that integrates metabolic control, inflammasome inhibition, inflammatory signaling modulation, and antioxidant defense activation (1, 15). Collectively, these mechanisms reshape microglial responses, reduce neuroinflammation, and protect neural tissues under ischemia, trauma, and neurodegeneration conditions. The regulatory cascade begins with SDH inhibition and the subsequent reduction in mitochondrial ROS production, which forms the metabolic basis for downstream immune regulation. This metabolic shift directly affects NLRP3 inflammasome activity, thereby limiting pyroptosis and the release of pro-inflammatory cytokines (14, 61). Concurrently, itaconate and its derivatives suppress the NF-κB signaling pathway, attenuating the transcriptional priming of inflammatory mediators (60). Finally, through the activation of the Nrf2 pathway and upregulation of downstream targets, such as heme oxygenase-1 (HO-1), itaconate strengthens antioxidant capacity and promotes cellular resilience (53) (**Figure 2**).

### 5.1 Inhibition of SDH and ROS production

The immunometabolic effects of itaconate are attributed to its ability to remodel mitochondrial metabolism. SDH is a metabolic enzyme that catalyzes the oxidation of succinate to fumarate in the TCA cycle. In addition to its role in the TCA cycle, SDH also contributes to reversing electron transport in mitochondria. As a component of complex II, SDH oxidizes the accumulated succinate, leading to the over-reduction of coenzyme Q. This drives electrons back into complex I, resulting in the generation of superoxide anions, which are the primary mitochondrial ROS. Excessive ROS subsequently activates inflammasomes, thereby promoting the secretion of pro-inflammatory mediators (62).

The inhibition of SDH by itaconate was first demonstrated in 1949 (63). Subsequent investigations by Lampropoulou et al. demonstrated that itaconate shares high structural homology with succinate, a natural substrate of SDH (18). This structural similarity allows itaconate to function as an endogenous competitive inhibitor, specifically occupying the catalytically active site of the enzyme. This noncovalent reversible binding effectively impedes the oxidation of succinate to fumarate, thereby directly suppressing the enzymatic activity of SDH (39, 64).

The inhibition of SDH activity precipitates significant metabolic reprogramming of mitochondrial pathways at the subcellular level. In the context of the electron transport chain (ETC), itaconate interrupts electron entry via Complex II, leading to disruption of the TCA cycle and subsequent intracellular succinate accumulation (65, 66). Crucially, this inhibitory action functions as a central regulatory mechanism to effectively restrict the generation of mitochondrial reactive oxygen species (mtROS). Under potent inflammatory stimulation (e.g., LPS induction), the unchecked oxidation of succinate by SDH, in the absence of itaconate-mediated inhibition, drives the hyper-reduction of the coenzyme Q (CoQ) pool and an aberrant elevation of mitochondrial membrane potential. These conditions compel reverse electron transport (RET) from CoQ back to complex I, resulting in the excessive production of superoxide, which contributes to inflammatory responses and tissue damage (67, 68). By competitively binding, to SDH, itaconate abrogates the RET pathway at its source, significantly attenuating pro-inflammatory mtROS production (69).

In summary, itaconate orchestrates the reprogramming of mitochondrial bioenergetics and redox metabolism through its primary action of SDH inhibition, establishing a mechanistic foundation for the downstream regulation of innate immune responses.

### 5.2 Inhibition of NLRP3 inflammasome activation

Building on the metabolic regulation of ROS, itaconate directly influences inflammasome activation. Inflammasomes are key components of the innate immune system that function as multiprotein complexes assembled through cytosolic pattern-recognition receptors. They recognize pathogen-associated molecular patterns (PAMPs), as well as damage-associated molecular patterns (DAMPs) (70). Among these, the NLRP3 inflammasome is one of the most well characterized. This inflammasome consists of the core sensor protein NLRP3, adaptor protein ASC, and mitotic kinase NIMA-related kinase 7 (NEK7), which are essential for its assembly and activation (8, 71).

Previous studies have demonstrated the importance of inhibiting the NLRP3 inflammasome in promoting neurological recovery after traumatic injury and ischemic stroke. Mechanistically, NLRP3 mediates the canonical pyroptotic pathway in microglia, which is characterized by cellular membrane perforation and disintegration, eventually resulting in cell lysis. Canonical pyroptosis is initiated by NLRP3 inflammasome assembly, followed by caspase-1 activation, gasdermin D (GSDMD) cleavage, pore formation, and subsequent release of the pro-inflammatory cytokines IL-1β and IL-18 (72). The formation of GSDMD pores disrupts osmotic balance, causing cellular swelling and lysis (73).

Itaconate and its derivatives suppress NLRP3 inflammasome activation through multiple interconnected mechanisms (**Figure 2**). Chief among these is the direct covalent modification of the NLRP3 protein at critical cysteine residues by derivatives such as 4-OI. Specifically, 4-OI alkylates Cysteine 548 (C548) residue in murine NLRP3. This alkylation creates steric hindrance that disrupts the essential interaction between NLRP3 and NEK7. As the binding of NEK7 is an obligate prerequisite for the conformational change and oligomerization of the NLRP3 sensor, this modification effectively arrests the assembly process at the initiation stage. Consequently, the failure of NLRP3 to oligomerize prevents the recruitment and polymerization of the adaptor protein apoptosis-associated speck-like protein containing a CARD (ASC), thereby blocking ASC speck formation (supramolecular signaling platforms). Without a functional ASC platform, the recruitment and autocatalytic cleavage of pro-caspase-1 are inhibited. This caspase-1 activation process blockade ultimately prevents the cleavage of gasdermin D (GSDMD) and the maturation of pro-inflammatory cytokines IL-1β and IL-18, thereby limiting pyroptosis and tissue damage (74, 75). Similarly, DMI curtails NLRP3 oligomerization and subsequent pyroptosis (76).

In addition to direct targeting, itaconate modulates inflammatory signaling pathways by inhibiting NF-κB activation. This downregulates the expression of NLRP3 and pro-inflammatory enzymes, such as caspase-1 (77). Itaconate also synergizes with inducible nitric oxide synthase (iNOS) and promotes toll-like receptor (TLR) ligand-induced tolerance, thereby restraining sustained NLRP3 inflammasome activation (61). As a TCA-cycle-derived metabolite that is highly upregulated in inflammatory macrophages, itaconate exemplifies the interplay between immunometabolism and innate immunity. It exerts immunoregulatory effects via widespread cysteine modification of target proteins, thereby dampening inflammasome activity (78, 79). Furthermore, emerging evidence indicates that itaconate influences autophagy and NLRP3 activation through pathways such as AMPK/mTOR, unveiling new avenues for metabolic interventions in inflammatory diseases (80). Therefore, by controlling mitochondrial ROS and NLRP3 assembly, itaconate acts as a key checkpoint for limiting pyroptosis. This establishes a mechanistic link with the broad suppression of pro-inflammatory transcription networks such as NF-κB.

### 5.3 NF-κB signaling pathway inhibition

Following inflammasome regulation, itaconate extends its influence to the transcriptional control of inflammation via the NF-κB pathway. IκB kinase β (IKKβ), a catalytic subunit of the IκB kinase (IKK) complex that also comprises IKKα and the regulatory scaffold NEMO (IKKγ), is indispensable in canonical NF-κB signaling (81). Itaconate and its derivatives, such as DMI and 4-OI, function as pivotal immunometabolic effectors that link cellular metabolism to innate immune responses (82). These compounds exert multi-level negative regulation of NF-κB signal transduction, primarily through the electrophilic alkylation of cysteine residues on critical signaling proteins (64).

Specifically, upstream of the canonical NF-κB activation pathway, emerging evidence suggests that itaconate derivatives—particularly DMI—directly target the core catalytic subunit of the IKK complex IKKβ via a Michael addition reaction. By covalently modifying specific cysteine residues on IKKβ (e.g., Cys412), DMI significantly impairs its kinase activity, thereby abrogating the phosphorylation and subsequent proteasomal degradation of the inhibitory protein IκBα. Consequently, NF-κB dimers are sequestered within the cytoplasm, preventing their nuclear translocation and inflammatory signaling initiation (40).

In addition to the direct inhibition of kinase activity, itaconate exerts a unique and selective fine-tuning mechanism for the transcription of NF-κB downstream target genes. Bambouskova et al. (2018) reported that the electrophilic stress induced by itaconate and its derivatives rapidly upregulates the expression of the transcription factor ATF3 (83). Rather than directly interfering with the DNA-binding capacity of NF-κB, ATF3 suppresses the protein synthesis of IκBζ—a critical nuclear co-activator of NF-κB—by targeting the 3’ untranslated region (3’UTR) of the Nfkbiz gene to regulate its translation efficiency (84). As IκBζ is indispensable for the transcriptional initiation of a specific subset of “secondary response genes” (e.g., IL-6, IL-12b), ATF3-IκBζ axis disruption results in the selective silencing of these pro-inflammatory cytokines while sparing “primary response genes” such as TNF-α. Notably, this regulatory mechanism operates independently of the Nrf2 pathway (85).

Additionally, itaconate promotes NF-κB degradation by regulating the global ubiquitination pattern, thereby further suppressing the production of inflammatory cytokines, such as TNF-α and IL-6 (86). As ROS are known activators of the NF-κB pathway, the reduction in ROS levels also indirectly attenuates NF-κB activation, thereby suppressing the pro-inflammatory response of microglia (11) (**Figure 2**). In addition, the antioxidant transcription factor Nrf2 has been demonstrated to be activated by itaconate and its derivatives, such as 4-OI (42, 53). Nrf2 functions as a major antagonist of NF-κB, and the activities of these two pathways are mutually antagonistic. The activation of Nrf2 not only dampens NF-κB signaling, but also reduces the production of pro-inflammatory mediators, including TNF-α and IL-6 (87). This mutual antagonism provides a mechanistic bridge toward the final layer of regulation, Nrf2-driven antioxidant defense, in which itaconate promotes cellular resilience beyond inflammation control.

### 5.4 Activation of the Nrf2 signaling pathway

As a culmination of its regulatory network, itaconate activates the Nrf2 signaling pathway, thereby reinforcing antioxidant and cell protection responses. Nrf2 is a key transcription factor that regulates the expression of a broad spectrum of antioxidant, detoxifying, and cytoprotective genes (88, 89). By coordinating these transcriptional programs, Nrf2 confers resistance to oxidative stress, inflammatory injury, and xenobiotic toxicity (90–92). In addition to its canonical role in redox homeostasis, Nrf2 modulates metabolic and immune responses, enabling neural cells to adapt to pathological stressors (92, 93). Given the complexity of its transcriptional and post-translational regulation, Nrf2 activity dysregulation has been closely linked to a variety of neurological disorders driven by oxidative stress and neuroinflammation (93–95) (**Figure 2**).

Recent studies have shown that itaconate and its derivatives, such as 4-OI and DMI, function as endogenous electrophiles that specifically target the KEAP1–Nrf2 axis. By undergoing Michael addition to alkylate distinct cysteine residues on the sensor protein KEAP1, most notably Cys151, Cys273, and Cys288, these compounds induce conformational changes that disrupt the assembly of the KEAP1–Cul3–Rbx1 E3 ubiquitin ligase complex (96, 97). This modification effectively abrogates the proteasomal degradation of Nrf2, thereby facilitating its nuclear accumulation (64, 98). Upon translocation to the nucleus, Nrf2 heterodimerizes with small Maf proteins and binds to antioxidant response elements (AREs) to orchestrate the transcription of a comprehensive cytoprotective gene network (97), including HO-1 (76), NADPH quinone oxidoreductase 1 (NQO1) (99), and glutathione biosynthesis genes such as the glutamate–cysteine ligase catalytic (GCLC) and glutamate–cysteine ligase modifier (GCLM) subunits. This transcriptional program restores redox homeostasis and suppresses ROS-dependent inflammatory cascades (100, 101). Consistent with this molecular framework, Ren et al. reported that DMI enhances Nrf2 expression in both the dorsal root ganglia and spinal cord tissues in a spinal nerve ligation model, whereas the analgesic effect of DMI is eliminated by the administration of the Nrf2 inhibitor ML385, confirming the central role of Nrf2 in this process (52). Furthermore, in models of spinal cord injury, itaconate-induced Nrf2 signaling in microglia not only attenuates inflammatory responses, but also promotes tissue preservation, reduces neuronal loss, and facilitates functional recovery (53). Similarly, in cerebral ischemia-reperfusion injury, exogenous itaconate enhances Nrf2-dependent transcriptional programs, thereby restoring the redox balance, limiting excitotoxic damage, and supporting neuronal survival (102, 103).

Among Nrf2 target genes, HO-1 represents a major effector of cytoprotection (104). Ischemic stroke induces *Irg1* expression in the microglia, whereas *Irg1* deletion suppresses HO-1 production and exacerbates tissue damage (105). Kuo et al. demonstrated that DMI administration compensated for the loss of the *Irg1*–itaconate axis, restored HO-1 expression, improved motor outcomes, and reduced mortality, underscoring the functional relevance of this pathway (106). In summary, these findings highlight Nrf2 activation as a convergent mechanism of itaconate action, integrating metabolic reprogramming, inflammasome suppression, and antioxidant defense into a unified protective response central to neuronal survival in CNS injury and disease.

## 6 Role of itaconate and its derivatives in several important central nervous system disorders

Itaconate and its derivatives have demonstrated therapeutic potential in a variety of CNS disorders, including cerebral ischemia, cerebral hemorrhage, and neurodegenerative diseases, such as AD and PD. Rather than merely modulating molecular signaling, recent preclinical studies have highlighted that these compounds deliver tangible functional improvements, such as reduced tissue damage, preservation of neuronal circuitry, and recovery of behavioral deficits. Collectively, itaconate and its derivatives are promising candidates for the treatment of CNS disorders because they translate metabolic control into physiological recovery (**Tables 2** & **3**).

**Table 2:** Role of itaconate and its derivatives in different diseases.

Disease	Target/signaling pathway	Cellular/biological effects	Therapeutic outcome	References
Cerebral Ischemia	1. Nrf2/HO-1 axis 2. SDH inhibition	1. Up-regulation of antioxidant enzymes 2. Reduction of mitochondrial ROS accumulation 3. Metabolic reprogramming driving M1-to-M2 microglial transition	1. Alleviation of neuroinflammation 2. Restoration of metabolic homeostasis and reduction of tissue damage 3. Improvement of motor outcomes and neurological deficits	(36, 102, 106)
Alzheimer’s Disease	1. NLRP3 inflammasome 2. Nrf2 pathway	1. Inhibition of inflammasome assembly and pyroptosis 2. Suppression of Aβ-induced oxidative stress. 3. Regulation of microglial metabolism toward anti-inflammatory phenotype	1. Mitigation of Aβ deposition and plaque accumulation 2. Improvement of cognitive function and memory deficits 3. Prevention of neuronal loss and cortical damage	(41)
Parkinson’s Disease	1. SDH & Complex II 2. Nrf2 pathway	1. Reduction of mitochondrial ROS generation 2. Attenuation of toxin (e.g., MPTP/rotenone)-induced neurotoxicity 3. Inhibition of pro-inflammatory M1 microglia polarization	1. Protection of dopaminergic neurons in the substantia nigra 2. Reduction of inflammation-induced neurodegeneration 3. Improved acute neurological prognosis and survival	(50, 107)
Cerebral Hemorrhage	1. KEAP1–Nrf2–CD36 axis 2. GPx4 alkylation	1. Enhancement of erythrophagocytosis by microglia 2. Inhibition of ferroptosis via GPx4 activation	1. Accelerated hematoma resolution 2. Prevention of secondary brain injury and neuronal apoptosis 3. Improved acute neurological prognosis and survival	(42, 108)

**Table 3:** Summary of preclinical studies of itaconate and its derivatives in CNS disorders.

Disease model	Compound	Dosage & administration	Animal/Strain	Core findings	Reference
Ischemic Stroke	DMI	20 mg/kg i.p., single dose immediately after reperfusion	C57BL/6 mice	Inhibited M1 microglial polarization; reduced infarct volume and neurological deficits; suppressed IL-1β expression.	(36)
Ischemic Stroke	DMI	25 mg/kg i.p., daily for 3 days post-surgery	*Irg1*-/-mice	Restored HO-1 expression; reduced mortality and brain edema; compensated for the loss of endogenous itaconate.	(106)
Alzheimer’s Disease	DMI	30 mg/kg i.p., daily for 2 months	APP/PS1 mice	Activated Nrf2/HO-1 pathway; reduced Aβ plaques and phosphorylated tau; improved spatial learning and memory.	(41)
Parkinson’s Disease	Itaconate	40 mg/kg i.p., daily for 7 days	C57BL/6 mice	Inhibited NLRP3 inflammasome activation; prevented dopaminergic neuron loss in substantia nigra; reduced locomotor impairment.	(50)
Multiple Sclerosis	DMI	80 mg/kg i.p., daily starting from day 7 post-immunization	C57BL/6 mice	Suppressed CNS infiltration of Th17 cells; reduced spinal cord demyelination; ameliorated clinical scores of paralysis.	(37)
Cerebral Hemorrhage	4-OI	50 mg/kg i.p., single dose 1h after ICH	C57BL/6 mice	Alkylated KEAP1 to upregulate CD36; enhanced hematoma clearance via microglial erythrophagocytosis; improved neurological function.	(42)
Cerebral Hemorrhage	4-OI	10–50 mg/kg i.p., daily for 3 days	C57BL/6 mice	Inhibited ferroptosis by alkylating GPx4; reduced iron-induced neuronal death and secondary brain injury.	(108)
Neuropathic Pain	4-OI	50 mg/kg i.p., daily for 7 days	C57BL/6 mice	Increased IL-10 expression in the spinal cord; inhibited astrocyte/microglia activation; attenuated mechanical allodynia.	(109)

### 6.1 Cerebral ischemia

Cerebral ischemia is a clinical condition with high morbidity and mortality, characterized by cerebrovascular obstruction that limits the blood supply to the brain, resulting in ischemic and hypoxic necrosis of local brain tissues. Itaconate and its derivatives have demonstrated significant functional benefits in ischemic stroke models, primarily manifested as reduced infarct volume, amelioration of neurological deficits, and preservation of BBB integrity.

In tMCAO mice, Zhang et al. reported that DMI prevented the toxic transition of peri-infarct microglia, which translated into a significant reduction in cerebral infarct volume and alleviated neurological deficit scores. They also reported that DMI administration effectively maintained BBB integrity and reduced brain edema (36). Similarly, Cordes et al. reported that itaconate facilitated the progressive recovery of mitochondrial respiratory function after reperfusion. This metabolic recovery directly limited tissue damage and improved cell survival rates in the ischemic penumbra (102). Furthermore, in 2021, Kuo et al. demonstrated that the administration of DMI to compensate for the loss of the *Irg1*–itaconate axis not only restored HO-1 expression, but also, and more importantly, significantly improved motor outcomes and decreased mortality rates in Irg1-/- stroke mice (106).

Collectively, these studies underscore the therapeutic potential of DMI in ischemic stroke and highlight its capacity to translate oxidative stress modulation into structural tissue protection and functional motor recovery.

### 6.2 Alzheimer’s disease

The most prevalent form of dementia, AD, is typified by aberrant amyloid-β (Aβ) plaque accumulation, neurofibrillary tangles, neuroinflammation, and neuronal death (110). Functionally, treatment with itaconate derivatives has been demonstrated to reduce the pathological plaque burden and reverse cognitive decline.

Specifically, in 2022, Xiong et al. demonstrated that DMI could rescue cognitive deficits in an APP/PS1 mouse model of AD. Behavioral testing revealed that DMI treatment significantly improved spatial learning and memory performance in the Morris Water Maze. Neuropathologically, this functional improvement was correlated with a marked reduction in Aβ plaque deposition in the cortex and hippocampus, as well as the prevention of neuronal loss (41).

Collectively, these findings highlight the therapeutic potential of DMI in AD, suggesting that it primarily alleviates neuroinflammation and cognitive deficits through Nrf2 pathway activation.

### 6.3 Parkinson’s disease

PD is the second most common progressive neurodegenerative disorder among older adults in the United States and is characterized by the presence of Lewy bodies and the loss or degeneration of dopaminergic neurons in the substantia nigra of the midbrain (111). In PD models, the administration of itaconate or its derivative 4-OI has been directly linked to the survival of dopaminergic neurons and restoration of motor coordination.

In 2022, Sun et al. reported that itaconate treatment effectively rescued tyrosine hydroxylase-positive (TH+) dopaminergic neurons in the substantia nigra from degeneration in both 1-methyl-4-phenylpyridinium (MPP+)-treated cellular models and 1-methyl-4-phenyl-1,2,3,6-tetrahydropyridine (MPTP)-induced PD mice. This cellular protection resulted in significant motor recovery, with treated mice showing improved coordination and reduced locomotor impairment in behavioral assessments (50). Supporting these findings, Xia et al. reported that 4-OI exerted microglia-dependent neuroprotective effects against rotenone- and MPP+-induced neurotoxicity in PD models, further confirming its ability to delay disease progression and preserve motor functions (107).

### 6.4 Cerebral hemorrhage

Cerebral hemorrhage is a life-threatening acute brain injury commonly caused by hypertension, vascular malformations, or anticoagulant use. Following hemorrhage, the perihematomal brain tissue undergoes an acute inflammatory response, leading to neuronal death, BBB disruption, and cerebral edema (112, 113). Structurally optimized derivatives, particularly 4-OI, protect neurons by enhancing microglial erythrophagocytosis, thereby mitigating iron-induced apoptosis and reducing secondary brain injury.

In 2024, Luo et al. found, in a mouse model of cerebral hemorrhage, that treatment with 4-OI significantly enhanced the phagocytic efficiency of microglia toward red blood cells. This led to a rapid reduction in hematoma volume and alleviated brain edema during the acute phase. Mechanistically, 4-OI alkylation of cysteine 155 on KEAP1 leads to the nuclear translocation of Nrf2 and transcriptional activation of the CD36 gene. Thus, the *Irg1*/itaconate axis enhances microglial phagocytosis by activating the KEAP1–Nrf2–CD36 axis (42). In the same year, Wei et al. found that itaconate prevented ferroptosis-induced neuronal death, thereby improving survival rates and long-term neurological prognosis after cerebral hemorrhage (108).

### 6.5 Other CNS disorders

Itaconate and its derivatives exert neuroprotective effects in various CNS disorders by modulating immune cell metabolism via the TCA cycle, which decreases pro-inflammatory cytokine release and mitigates neuroinflammation. Simultaneously, they enhance antioxidant defenses through the Nrf2 pathway, protecting neurons and glial cells from oxidative damage, highlighting itaconate as a potential therapeutic target for CNS disorders.

Sun et al. (2022) demonstrated that both endogenous and exogenous itaconate exerted significant analgesic effects on neuropathic pain. The administration of 4-OI increased IL-10 expression in the spinal cord and activated the STAT3/endorphin signaling pathway. This analgesic effect was significantly diminished in IL-10-deficient mice, underscoring the pivotal role of IL-10. Further mechanistic analysis revealed that the neuronal activation of the Nrf2 pathway was responsible for 4-OI-induced IL-10 upregulation, suggesting a neuroimmune crosstalk mechanism in pain modulation (109).

In addition to neuropathic pain, accumulating evidence has linked itaconate to infectious CNS diseases. *Toxoplasma gondii* infection disrupts the ACOD1/itaconate axis, leading to excessive pro-inflammatory gene expression and aberrant microglial activation. He et al. reported that treatment with DMI significantly alleviated *T. gondii*-induced cognitive deficits (114). These improvements were accompanied by the restoration of synaptic ultrastructure, decreased pro-inflammatory microglial clustering, and enhanced behavioral performance, highlighting the potential of DMI in controlling parasite-induced neuroinflammation.

Other infections also implicate the itaconate pathway in neuroprotection. In 2023, Wu et al. demonstrated that *Neisseria gonorrhoeae* infection induced behavioral impairments in mice, which were associated with neuroinflammation, synaptic ultrastructural damage, and metabolic dysregulation in the prefrontal cortex. Metabolic profiling revealed a shift toward glycolysis and fatty acid oxidation, along with impaired Krebs cycle function and disruption of the ACOD1–pyruvate axis. Importantly, DMI treatment effectively attenuated these pathological changes and prevented both neuroinflammation and behavioral deterioration (115). Similarly, in 2024, Tomalka et al. reported that the cerebrospinal fluid levels of itaconate were significantly reduced in patients with tuberculous meningitis, with lower concentrations negatively correlated with pro-inflammatory cytokines and chemokines, suggesting that endogenous itaconate may act as an intrinsic brake on CNS inflammation (116).

In addition to infection-related conditions, 4-OI has been investigated in autoimmune neuroinflammatory disorders. Li et al. investigated the effects of 4-OI on NLRP3 inflammasome activation in monocytes and macrophages via the *Irg1*–itaconate–NLRP3 pathway using an *in vitro* model of neuromyelitis optica spectrum disorder (NMOSD), a rare inflammatory astrocytic disease of the central nervous system. Their study revealed that *Irg1* expression was upregulated in patients with acute NMOSD, with monocytes showing significantly elevated NLRP3 inflammasome activation and pro-inflammatory mediator levels. Importantly, 4-OI effectively protected patients with acute NMOSD, highlighting its potential as a therapeutic strategy against inflammatory CNS disorders (117).

## 7 Potential research areas

Although recent research has shed light on the function of itaconate in regulating inflammation and microglial metabolism, several important questions remain to be answered, opening up new research directions.

### 7.1 Elucidation of molecular mechanisms

Exploring the chemical mechanisms through which itaconate and its derivatives regulate microglial activation remains a central area for future research. Although itaconate is known to attenuate inflammation via activation of the Nrf2 pathway, its synergistic interactions with other signaling cascades (including NF-κB inhibition, inflammasome modulation, and metabolic reprogramming) are still poorly defined. Moreover, the precise mechanism by which the *Irg1*/itaconate pathway regulates microglial energy metabolism (e.g., glycolysis and oxidative phosphorylation) and its link to pro-inflammatory phenotypes requires further clarification (15). Although itaconate has been reported to affect mitochondrial function by inhibiting SDH, its detailed crosstalk with pathways such as Nrf2/KEAP1 and the NLRP3 inflammasome remains to be elucidated (1, 10).

Considering these knowledge gaps, future studies should integrate advanced methodologies to elucidate the multidimensional regulatory roles of itaconate (118). Approaches such as single-cell RNA sequencing, spatial transcriptomics, and proteomic profiling, combined with CRISPR/Cas9-based gene editing and bioinformatics, are essential for clarifying how itaconate interfaces with metabolic and inflammatory signaling in microglia (119–121). This work will provide mechanistic insights into unresolved questions, including the context-specific regulation of energy metabolism and its interaction with canonical inflammatory pathways, thereby advancing the translational potential of itaconate in CNS disorders.

### 7.2 Development of novel derivatives

Despite its potent biological activity, the clinical translation of itaconate is limited by its poor pharmacokinetics, low bioavailability, and restricted BBB penetration. Consequently, the development of novel itaconate derivatives has become a critical research focus (10). Strategic structural modifications enhance lipid solubility and stability, whereas prodrug design enables tissue-specific release. Furthermore, cutting-edge drug-delivery systems, including polymer microspheres, liposomes, and nanoparticle drug-carrying systems, can significantly enhance therapeutic efficacy and medication targeting. For instance, toward the goal of intracellular itaconate delivery, degradable polyester polymer (poly(dodecyl itaconate))-based itaconate polymer microparticles (IA-MPs) can be generated using an emulsion method to improve their distribution and long-lasting effects on the neurological system (122). These strategies decrease side effects and increase the efficacy of medication.

### 7.3 Exploration of disease-specific applications

Several neurological disease models, including ischemic stroke, PD, and AD, have been used to provide preliminary evidence for the therapeutic potential of itaconate and its derivatives (41, 102, 107). However, its relevance to other major neurological conditions, such as multiple sclerosis (MS), traumatic brain injury (TBI), and glioblastoma, remains unknown. Future research should focus on elucidating whether itaconate reduces axonal damage and demyelination in MS models, potentially by preventing the production of inflammatory factors and microglial hyperactivation. Studies should also examine the mechanisms by which it suppresses oxidative stress and inflammation and promotes repair during the acute phase of TBI. Additionally, studies on malignant gliomas should focus on determining whether metabolic reprogramming can stop tumor invasion and progression by adjusting immune cell activity in the tumor microenvironment.

The elucidation of the disease-specific mechanisms of action of itaconate will benefit from multimodal imaging analysis, multi-omics studies, and the development of disease-specific preclinical animal models. Tools for the real-time monitoring of itaconate dynamics (such as the BioITA biosensor) can also be developed to analyze metabolic changes in subcellular localization (123).

### 7.4 Clinical translation and safety assessment

Despite the safety of itaconate and its derivatives as supported by current preclinical research, there are several obstacles to clinical translation. The efficacy of itaconate in preclinical models must be validated in human cells or organoids; however, species differences may influence the results (62). More thorough clinical trials are required to evaluate the pharmacokinetic characteristics, safety, and efficacy of these drugs in humans. Additionally, long-term studies should focus on potential off-target effects, such as effects on other cells within the CNS or interference with other metabolic pathways. Moreover, the long-term safety and tolerability of itaconate derivatives remain unclear, particularly regarding their potential interference with brain homeostasis. High concentrations of itaconate or its derivatives may adversely affect glucose and glutathione metabolism in astrocytes (114, 124).

Furthermore, dose optimization studies and the investigation of optimal therapeutic windows are essential for balancing efficacy and safety. Finally, guided by the principles of precision medicine, patients’ genetic backgrounds and disease characteristics may help in identifying individuals who are most likely to benefit, enabling the development of tailored treatment regimens that maximize therapeutic efficacy and minimize adverse effects.

### 7.5 Understanding the role of itaconate in CNS metabolism

Itaconate plays a complex and context-dependent role in CNS metabolism and neuroinflammation; however, its precise mechanism of action remains unclear. By inhibiting SDH and potentially fumarate hydratase, itaconate alters the flux of the TCA cycle, resulting in the accumulation or depletion of critical intermediates such as succinate and fumarate (125, 126). These changes can influence inflammatory and antioxidant signaling, including succinate-driven HIF-1α activation and fumarate-mediated Nrf2 stabilization. While protective effects have been demonstrated, for example, 4-OI restoring microglial metabolic homeostasis, suppressing NLRP3 inflammasome activation, and improving neurological outcomes after traumatic brain injury (15), potential detrimental effects have also been reported. Excessive succinate accumulation may promote pro-inflammatory cytokine release, fumarate depletion may impair Nrf2-dependent cytoprotection, and *in vitro* studies have indicated possible cell-type-specific metabolic interference. However, current evidence regarding the itaconate–succinate–fumarate axis in the CNS is limited, and the direct effects of itaconate on neurons and astrocytes remain poorly understood (127).

Future research should focus on systematically characterizing the dynamic interplay between itaconate and mitochondrial metabolites under various pathological conditions (128). Advanced techniques, such as metabolic flux analysis, multiphoton microscopy, and activity-based metabolic probes, can provide spatiotemporal insights into their distribution and activity. In parallel, integrative approaches combining metabolomics, functional genetics, and disease-specific models are essential for clarifying how the immunomodulatory properties of itaconates can be harnessed therapeutically without compromising metabolic adaptability (129, 130). Ultimately, delineating the context-dependent determinants of the protective and detrimental effects of itaconate is crucial for translating itaconate-based strategies into effective interventions for CNS disorders.

### 7.6 Combination of precision medicine and personalized treatment

From the perspectives of individual genomics, epigenomics, and metabolomics, future research on itaconate and its derivatives can integrate precision medicine principles by examining individual variations in therapeutic effects (128, 131). For instance, targeting the distinct metabolic profiles of microglia under different disease states may enable the development of more precise and personalized pharmacological strategies (15, 60, 132, 133). Furthermore, leveraging artificial intelligence and machine learning to integrate multi-omics data with clinical features can enhance drug screening and efficacy prediction, thereby accelerating clinical translation (86, 134).

In conclusion, bridging the gap between fundamental research and clinical applications is the main goal of future studies on itaconate and its derivatives. Thorough investigation of molecular mechanisms, optimization of derivative design, exploration of disease-specific applications, and rigorous assessment of clinical safety and efficacy are essential for fully realizing the therapeutic potential and pioneering novel treatments for neurological disorders. Continued advancements in research tools and methodologies provide strong justification for optimism regarding the role of itaconate in addressing neuroinflammation and metabolic dysregulation.

## 8 Conclusion

Itaconate has emerged as a pivotal immunometabolite linking metabolic reprogramming to microglial activation and neuroinflammation. By targeting multiple signaling pathways, including the inhibition of succinate dehydrogenase and mitochondrial ROS generation, suppression of NF-κB-driven transcription, blockade of NLRP3 inflammasome assembly, and activation of the Nrf2–HO–1 antioxidant axis, itaconate and its derivatives orchestrate a broad regulatory network that restrains neuroinflammation and enhances cellular resilience. These concerted actions attenuate microglial cytotoxicity, promote a shift toward reparative phenotypes, and provide neuroprotection in diverse CNS disorders. Preclinical studies have consistently demonstrated beneficial effects on ischemic stroke, AD, PD, and cerebral hemorrhage, as well as in neuropathic pain, infectious encephalitis, and autoimmune demyelination models. Collectively, these findings highlight itaconate as a promising therapeutic agent for modulating microglial metabolism in CNS diseases.

Despite these advances, several challenges have impeded their translation into clinical practice. The highly polar nature of itaconate limits BBB penetration, necessitating reliance on structural derivatives, such as DMI and 4-OI, which offer improved stability and permeability, but require further safety validation. Moreover, the pleiotropic mechanisms of action, while advantageous for broad immunoregulation, complicate the precise dissection of target specificity and increase the possibility of off-target metabolic effects in non-microglial cells. Current knowledge is largely derived from rodent models, and whether these findings translate to human neuropathology remains uncertain. Rigorous pharmacokinetic profiling, dosing regimen optimization, and disease-specific therapeutic window identification are essential prerequisites for clinical application. In addition, the heterogeneity of microglial phenotypes across disease contexts underscores the need for precise strategies that integrate patient-specific metabolic signatures with therapeutic interventions.

In summary, the itaconate–*Irg1* axis represents a central metabolic checkpoint in microglial regulation that bridges innate immunity, oxidative stress control, and tissue repair. The therapeutic potential of itaconate and its derivatives lies in their ability to simultaneously recalibrate neuroinflammatory signaling and restore metabolic homeostasis. Future research should focus on delineating the multidimensional molecular mechanisms, developing next-generation derivatives with optimized pharmacological properties, and conducting translational studies to address their long-term efficacy and safety in humans. These efforts will help in determining whether modulation of the itaconate pathway can be harnessed as a viable clinical strategy for treating neuroinflammatory and neurodegenerative diseases.
